# Neuroprotective effects of resveratrol and epigallocatechin gallate polyphenols are mediated by the activation of protein kinase C gamma

**DOI:** 10.3389/fncel.2013.00281

**Published:** 2013-12-26

**Authors:** Caroline Menard, Stéphane Bastianetto, Rémi Quirion

**Affiliations:** ^1^Laboratory of Neuroendocrinology of Aging, Centre Hospitalier de l'Université de Montréal Research CenterMontreal, QC, Canada; ^2^Department of Medicine, University of MontrealMontreal, QC, Canada; ^3^Douglas Mental Health University Institute, McGill UniversityMontreal, QC, Canada; ^4^Department of Psychiatry, McGill UniversityMontreal, QC, Canada

**Keywords:** PKC, polyphenols, neuroprotection, resveratrol, epigallocatechin gallate, hippocampal cultured cells

## Abstract

Polyphenols such as epigallocatechin gallate (EGCG) and resveratrol have received a great deal of attention because they may contribute to the purported neuroprotective action of the regular consumption of green tea and red wine. Many studies, including those published by our group, suggest that this protective action includes their abilities to prevent the neurotoxic effects of beta-amyloid, a protein whose accumulation likely plays a pivotal role in Alzheimer's disease. Moreover, the scavenging activities of polyphenols on reactive oxygen species and their inhibitory action of cyclooxygenase likely explain, at least in part, their antioxidant and anti-inflammatory activities. Besides these well-documented properties, the modulatory action of these polyphenols on intracellular signaling pathways related to cell death/survival (e.g., protein kinase C, PKC) has yet to be investigated in detail. Using rat hippocampal neuronal cells, we aimed to investigate here the effects of EGCG and resveratrol on cell death induced by GF 109203X, a selective inhibitor of PKC. The MTT/resazurin and spectrin assays indicated that EGCG and resveratrol protected against GF 109203X-induced cell death and cytoskeleton degeneration, with a maximal effect at 1 and 3 μM, respectively. Moreover, immunofluorescence data revealed that cells treated with these polyphenols increased PKC gamma (γ) activation and promoted neuronal interconnections. Finally, we found that the protective effects of both polyphenols on the cytoskeleton and synaptic plasticity were mediated by the PKCγ subunit. Taken together, the results suggest that PKC, and more specifically its γ subunit, plays a critical role in the protective action of EGCG and resveratrol on neuronal integrity.

## Introduction

It is well established that the regular consumption of fruit, vegetables, and beverages such as green tea and red wine (in moderation) reduces the risk of developing age-related neurological disorders such as Alzheimer's disease (AD) (Orgogozo et al., 1997; Truelsen et al., 2002; Luchsinger et al., 2004; Dai et al., 2006). Polyphenols present in high amounts in fruit, vegetables, tea, and red wine likely contribute to their beneficial effects.In support of this hypothesis, two follow-up studies reported that regular consumption of polyphenols was inversely correlated with the risk of dementia and cognitive decline (Commenges et al., 2000; Letenneur et al., 2007). Moreover, *in vitro* and animal studies reported that epigallocatechin gallate (EGCG), the most abundant polyphenol present in green tea, and resveratrol, a stilbene enriched in red wine, exerts neuroprotective actions against the toxicity induced by β-amyloid (Aβ) (Choi et al., 2001; Han et al., 2004; Marambaud et al., 2005; Rezai-Zadeh et al., 2005; Bastianetto et al., 2006; Haque et al., 2008; Lee et al., 2009), a protein whose accumulation plays a pivotal role in AD-related cognitive symptoms (Selkoe, 2000).

The modulation of intracellular effectors has been proposed to explain, at least partly, the effects of polyphenols in neurodegenerative processes. Among other effects, we have shown that protein kinase C (PKC) enzymes are involved in the neuroprotective effect of resveratrol against Aβ-induced neurotoxicity (Han et al., 2004; Bastianetto et al., 2007), while other groups have suggested that EGCG promotes the release of nonamyloidogenic soluble precursor through a PKC-dependent pathway (Levites et al., 2003). Moreover, Levites et al. (2002) have shown that activation of PKC by EGCG is linked to cell survival in Parkinson's disease, suggesting that PKC plays an important role in the neuroprotective action of theses polyphenols.

Recently, among the 12 isoforms of PKC (Newton, 1997), our group has reported that gamma (γ) activity is linked to successful cognitive aging (Menard and Quirion, 2012), while another group found that protein levels of PKCγ are lower in an AD mouse model (Dehvari et al., 2007). It is thus hypothesized that the purported cognitive enhancing properties of polyphenols in memory-impaired animals (Cherniack, 2012) could be due to their stimulatory effects on PKCγ activity. Accordingly, we investigate here the effects of resveratrol and EGCG on PKCγ activation in hippocampal neuronal cells exposed to a broad spectrum inhibitor of PKC known as dihydrochloride3-{1-[3-(dimethylamino) propyl]-1H-indol-3yl}-4-(1H-indol-3-yl)-1H-pyrrole-2,5-dione (GF 109203X). Both compounds prevent GF 109203X-induced neuronal death and the disruption of cytoskeleton organization. Interestingly, resveratrol and EGCG increase not only PKCγ (Thr674) phosphorylation, but also the expression of the kinase, suggesting an active role for this enzyme in neuronal cell survival promoted by these polyphenols.

## Material and methods

### Material

Materials used for cell cultures were obtained from Invitrogen-Gibco BRL (Burlington, Ontario, Canada). Resveratrol, EGCG and other chemicals were purchased from Sigma Chemical Co. (Oakville, Ontario, Canada). All drugs were freshly prepared on the day of the experiment in a final concentration of either ethanol or DMSO not exceeding 0.01%. These solvents at 0.01% (v/v) have no effect by themselves on cell survival (data not shown).

### Neuronal hippocampal cell cultures

Enriched rat hippocampal cell cultures were prepared from E19 fetuses obtained from Sprague-Dawley rats (Charles River Canada, St-Constant, Quebec, Canada) as described previously (Bastianetto et al., 2000). Animal care complied with protocols and guidelines of the McGill University Animal Care Committee and the Canadian Council for Animal Care. Hippocampal cells were plated at day 0 at a density of approximately 12 × 10^4^ viable cells per well in 96-well plates. In brief, hippocampal neuronal cells were grown in Dulbecco's modified Eagles medium (D-MEM) high glucose supplemented with 20 mM KCl, 15 mM HEPES and 1% (v/v) serum-free growth medium N2 (final composition: 5 μg/ml insulin, 100 μM putrescine, 20 nM progesterone, 1.0 μg/ml transferrin, 30 nM selenium), and maintained at 37°C in a 95% air/5% CO_2_ humidified atmosphere. Medium was removed at day 3 and replaced with the same medium until the day of experiment (day 6).

### GF 109203X-induced toxicity

GF 109203X (dihydrochloride3-[1-[3-(dimethylamino) propyl]-1H-indol-3yl]-4-(1H-indol-3-yl)-1H-pyrrole-2,5-dione) is a potent and selective inhibitor of PKC (Toullec et al., 1991) that has been shown to produce neuronal hippocampal cell death (Han et al., 2004). Briefly, cells were incubated in HEPES-buffered DMEM high-glucose medium and co-treated with GF 109203X (5 μ M) and either EGCG (1–10 μ M) or resveratrol (1–10 μ M). After a 24 h incubation period, cell viability was determined using the MTT and resazurin colorimetric assays (see below).

### Assessment of neuronal survival

Neuronal survival was estimated using MTT [3-(4,5-dimethylthiazol-2-yl)-2,5-diphenyl tetrazolium bromide], a dye that measures the mitochondrial activity of living cells (Bastianetto et al., 2000; Zhang et al., 2004). Cell survival was measured in parallel with resazurin, a widely used indicator of cell viability in mammalian cell cultures (Anoopkumar-Dukie et al., 2005). The resazurin assay is based on the ability of viable, metabolically active cells to reduce resazurin to resorufin and dihydro-resorufin. Toxic insults that impair cell viability affect the capacity to reduce resazurin, and the rate of dye reduction is directly proportional to the number of viable cells present (Vega-Avila and Pugsley, 2011). Cell survival estimated by the MTT and resazurin assays was spectrophotometrically determined at 570 nm. Regarding resazurin assay, background optical density (OD) at 600 nm was subtracted from OD at 570 nm for optimal result. Colorimetric assays were performed using a micro-plate reader (Bio-Tek Instruments® Inc., Ville St-Laurent, Quebec, Canada).

### Immunofluorescence

On day 0, hippocampal neurons were plated on poly-d-lysine (25 μ g/mL)-coated 12 mm glass coverslips (Fisher, Nepean, On, Canada) placed in multiwell plates and grown in the same medium as described above. On day 3, the medium was removed, hippocampal cells were washed in PBS (pH 7.4) for 5 min, then fixed in 4% paraformaldehyde. After several PBS washes, cells were permeabilized with 0.2% Tween 20 in PBS for 10 min at room temperature, then processed for immunofluorescence labeling. In brief, sections were first incubated in 10% normal goat serum diluted in 0.1 M PBS with 0.05% Tween 20 (PBST) and 1% BSA for 60 min at room temperature, followed by overnight incubation with primary antibodies at 4°C in a solution of 1% serum and 1% BSA in 0.1 M PBST. The anti-rabbit PKCγ (Thr674) and PKCγ antibodies were purchased from Abcam (Cambridge, MA, USA) and used at 1/100 dilution. To reveal the cytoskeleton structure, a spectrin antibody (1/250 dilution, produced in mouse) was purchased from Cell Signaling (Danvers, MA, USA). After 3 washes in PBS, sections were incubated with corresponding secondary antibodies (1:500, Invitrogen, Carlsbad, CA, USA) conjugated with Alexa Fluor 488 or Alexa Fluor 568 in 1% BSA in PBST for 2 h at room temperature in the dark. Sections were washed three times with PBS for 5 min each in the dark. Nuclei were stained with Hoechst solution (2 μg/ml, Invitrogen, Carlsbad, CA, USA) for 5 min and subsequently the sections were washed and coverslipped with Fluoromount-G (Southern Biotech, Birmingham, AL, USA). Pictures were taken at 40x magnification with an Axio Observer microscope with Apotome (Carl Zeiss MicroImaging GmbH, Germany).

### Statistical analyses

OD values reflecting MTT and resazurin reduction were proportional to the number of viable cells. The OD of the control group (vehicle alone) was regarded as 100%. The rate of surviving cells treated with various drugs was expressed as a percentage of control groups. Statistical analysis was performed using One-Way ANOVA followed by a Dunnett's multiple comparison test, with *p* < 0.05 being considered statistically significant.

## Results

### Effects of resveratrol and EGCG against toxicity induced by GF 109203X

**Figure 1:**
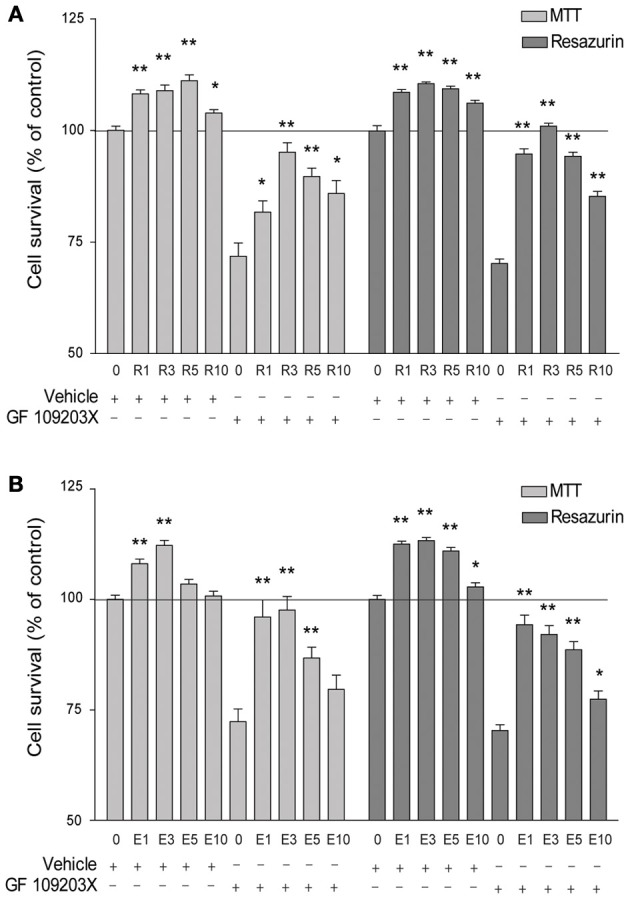
**Resveratrol (R) and epigallocatechin gallate (E) increase hippocampal cell survival, whether exposed or not to a PKC inhibitor. (A)** Cell survival is significantly enhanced by resveratrol treatment in comparison with the vehicle alone, with a maximal effect at 3 μM, as assessed by MTT and resazurin assays. The selective PKC inhibitor GF 109230X (5 μM) induces significant cell death that is rescued by co-treatment with resveratrol, these effects being maximal at 3 μM. **(B)** Similarly, cell survival is increased by EGCG treatment alone, with a maximal effect at 3 μM. Cell death produced by GF 109230X (5 μM) can be attenuated by EGCG, with a maximal effect at 1 μM. Data represent mean ± SEM from three (resazurin assay) to four (MTT assay) separate cultures and are expressed as percentages of the vehicle alone. Values were compared to the vehicle or GF 109203X alone for statistical analysis, ^*^p < 0.05, ^**^p < 0.01.

As described previously (Han et al., 2004), a 24 h treatment with the PKC inhibitor GF 109203X (5 μM) resulted in about 30% hippocampal neuronal cell death, as monitored using MTT and resazurin colorimetric assays (Figures 1A,B). Cell death was strongly reduced, in a concentration-dependent manner, in the presence of resveratrol with maximal effect obtained at 3 μM (Figures 1A). The effect of resveratrol was shared by EGCG, which offered maximal and almost complete protection at 1 μM, as estimated by both colorimetric assays (Figures 1B). Moreover, 24 h exposure to either resveratrol or EGCG in the initial medium (i.e., without GF 109203X) exerted a protective and maximal effect *per se* at 1–3 μM (MTT and resazurin), and higher concentrations tended to be less effective (Figures 1A,B).

### Polyphenols activate PKCγ enzyme, increase neuronal interconnections and prevent cytoskeleton degeneration

**Figure 2:**
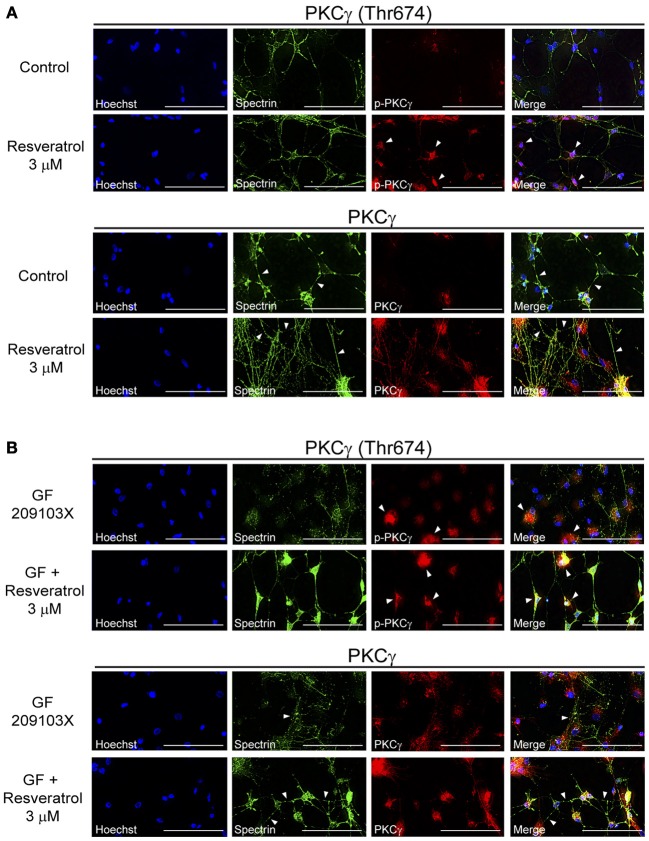
**Resveratrol treatment increases PKCγ activation, neuronal interconnections and maintains cytoskeleton integrity. (A)** Application of resveratrol (3 μM) activated PKCγ by phosphorylation at threonine 674 residue, increased PKCγ expression and neuronal interconnections in hippocampal cells. **(B)** Treatment with the PKC inhibitor GF 109203X (5 μM) disrupted the cellular cytoskeleton, as shown by spectrin immunofluorescence, and activation of PKCγ by resveratrol prevented this neurodegenerative process. Scale bar is set at 100 μm. Pictures are representative of average observations from three separate culture preparations.

Neuroprotective effects have been associated with PKCγ activation and signaling (Zhang et al., 2011) but have not yet been explored for polyphenols. Using co-immunofluorescence, we evaluated the effects of resveratrol and EGCG treatments on PKCγ activation and cell morphology. Indeed, PKCγ interact with proteins associated with neurite elongation, formation and maintenance of synaptic contacts (Zhang et al., 2011). As shown in Figure 2A, resveratrol (3 μM) increased both PKCγ (Thr674) phosphorylation and expression in hippocampal cultured cells. Moreover, cells treated with this polyphenol showed higher interconnections, as revealed by spectrin staining and indicated by arrows (Figure 2A). On the other hand, cells exposed to GF 109203X were characterized by altered synaptic connexions and cytoskeleton degeneration (Figure 2B). Few cells showed PKCγ activation, suggesting a compensatory mechanism to reduce cell death. PKCγ was phosphorylated on the Thr674 residue in multiple hippocampal cells treated with resveratrol following PKC inhibition (Figure 2B).

**Figure 3:**
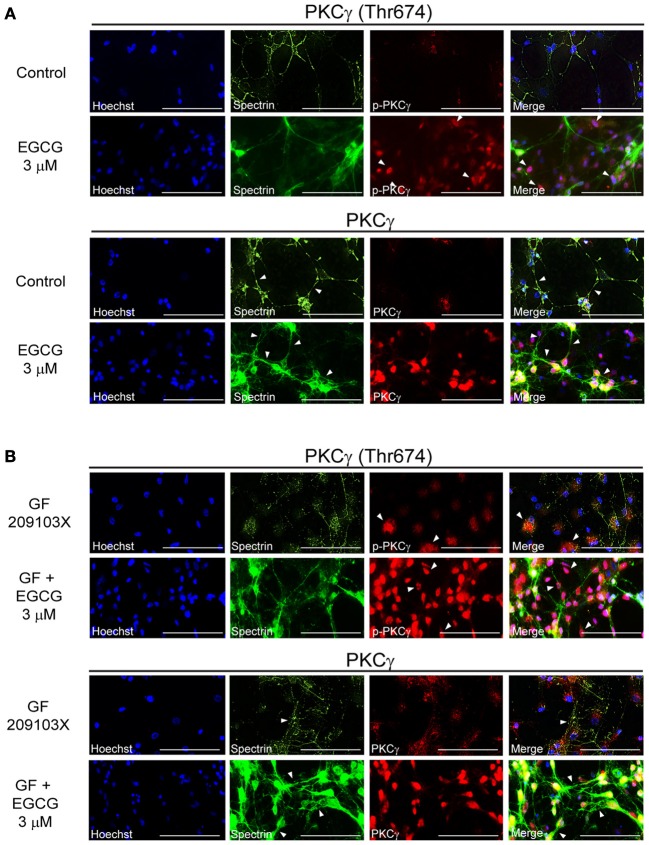
**EGCG enhances synaptic plasticity through PKCγ activation. (A)** EGCG (3 μM) treatment increased PKCγ expression, phosphorylation at the Thr674 site and facilitated neuronal interconnections. **(B)** This polyphenol can also prevent cytoskeleton neurodegeneration induced by GF 109203X (5 μM) by modulating PKCγ level and activity. Scale bar is set at 100 μm. Pictures are representative of average observations from three separate culture preparations.

Like resveratrol, EGCG (3 μM) alone seemed to promote neuronal interconnections and clearly increased PKCγ phosphorylation (Figure 3A). PKCγ enzymes were seen most prominently around the nuclei of cells treated with EGCG (Figure 3A), while it was evenly distributed in the cytoplasm after resveratrol application (Figure 2A). EGCG strongly increased PKCγ activity in GF109203X-treated cells and prevented cytoskeleton degeneration (Figure 3B).

## Discussion

In this study, EGCG and resveratrol, two active ingredients found in tea and wine respectively, were shown to, concentration (low μM)-dependently, protect against GF109203X-induced toxicity in cultured hippocampal neurons. These findings confirm and extend previous studies suggesting that the neuroprotective actions of resveratrol and EGCG involve the modulation of kinases such as PKC and cell death mediators (Miloso et al., 1999; Bastianetto et al., 2007; Bastianetto and Quirion, 2010; Bastianetto et al., 2011; Zhao et al., 2011).

Earlier, our group showed that resveratrol can reduce PKCδ phosphorylation in cultured hippocampal neuronal cells exposed to Aβ, suggesting that PKCδ is likely involved in resveratrol-mediated protection against Aβ-induced neurotoxicity (Han et al., 2004). We report here on the potential role of another isoform, PKCγ, in the neuroprotective effects of resveratrol and EGCG. It is well established that this isoenzyme is involved in synaptic development and neuronal plasticity. Indeed, PKCγ has been shown to interact with proteins associated with neurite elongation and formation, and the maintenance of synaptic contacts (Zhang et al., 2011). Moreover, it has been reported that PKCγ levels decline after ischemic preconditioning (Shamloo and Wieloch, 1999), and a down-regulation of PKCγ membrane translocation may be involved in a mouse model of hippocampal cell death mediated by oxygen-glucose deprivation (Liu et al., 2008). Finally, insulin-mediated inhibition of cortical neuronal necrosis following hypoxia has been reported to be associated with the translocation of PKCγ (Hamabe et al., 2005).

Both polyphenols increased PKCγ (Thr674) phosphorylation and its expression in presence of the PKC inhibitor. These effects occurred in the same concentration range as those protecting neuronal cells, suggesting an involvement of this PKC isoform in the neuroprotective action of the polyphenols. This hypothesis is supported by the finding that both polyphenols were able to prevent altered synaptic connexions and cytoskeleton degeneration induced by GF 109203X. Moreover, in the absence of the toxic agent, EGCG and resveratrol *per se* increased cell survival, accompanied by stimulation of PKCγ phosphorylation and maintenance of cytoskeleton architecture and neuronal plasticity. The effect of resveratrol on PKCγ was somewhat surprising, since we previously reported a lack of effects of resveratrol on PKCγ phosphorylation (Han et al., 2004). This apparent discrepancy may be explained by the concentrations used in the two studies (20 μM vs 3 μM in the present study).

These findings are of particular interest, since it is known that cytosolic PKCγ interacts with synapsin, a protein associated with synaptic vesicle release, which also binds with high affinity to actin, an essential component of the architecture of the cytoskeleton (Zhang et al., 2011). PKCγ can regulate cytoskeleton assembly (Rosenberg and Ravid, 2006) and has been reported to be actively involved in glutamate receptor trafficking, suggesting an important role for this kinase in synaptic development and plasticity (Patten and Ali, 2009). N-methyl-d-aspartate (NMDA) receptors are known to control dendritic spine motility, synaptogenesis and synapse stabilization (Gambrill and Barria, 2011). The stimulation of both ionotropic and metabotropic glutamate receptors can activate PKCγ, thus facilitating intracellular signaling and gene expression associated with learning and memory processes (Codazzi et al., 2006). Recently, (Huang et al., 2012) reported that reduction of the PKCγ-ERK (extracellular signal-regulated protein kinase) signaling pathway activation was involved in hippocampal neurodegeneration and persistent learning and memory impairments induced by ketamine, a NMDA receptor antagonist. Interestingly, the present results suggest that resveratrol and EGCG can facilitate hippocampal plasticity and interconnections through PKCγ activation, a process that could be associated with several higher brain functions, and that these polyphenols also prevent the suppression of PKCγ phosphorylation associated with hippocampal neurodegeneration (Huang et al., 2012).

Several missense mutations in the PKCγ gene have been found in spinocerebellar ataxia type 14 (SCA14), an autosomal dominant neurodegenerative disease (Yamamoto et al., 2010; Ueda et al., 2013). Mutant PKCγ kinases act as a dominant negative regulator on wild-type PKCγ enzymes, disrupting synaptic pruning, plasticity and transmission (Shuvaev et al., 2011). Mutant cytosolic PKCs have a tendency to aggregate, resulting in neuronal cell death (Seki et al., 2005; Asai et al., 2009). Interestingly, Yamamoto et al. (2010) reported that stimulation of autophagy provoked by treatment with rapamycin can promote the degradation of mutant PKCγ enzymes, while normal kinases are not affected. Moreover, aggregate formation and the cytotoxicity induced by mutant γPKCs has been reported to be inhibited in SH-SY5Y cells by congo red, a dye known to inhibit amyloid oligomers and fibril formation of misfolded proteins (Seki et al., 2010). Various groups, including ours, have shown that EGCG and resveratrol can stimulate autophagy in various types of cells, including macrophages (Li et al., 2011; Kim et al., 2013; Pallauf and Rimbach, 2013), and prevent the formation of Aβ oligomers and fibrils (Bastianetto et al., 2006, 2008; Cheng et al., 2013), suggesting that polyphenols may be beneficial in the treatment of SCA14 patients.

The concentrations of EGCG (165–275 μ M) and resveratrol (19–34 μ M) present in tea extracts and red wine (Sato et al., 1997; Wang et al., 2003; Del Rio et al., 2004; Hashim et al., 2013) are much higher than those required to produce their *in-vitro* protective effects as seen in the present study. Although polyphenols such as EGCG and resveratrol undergo significant metabolism and conjugation during absorption in humans (Goldberg et al., 2003; Spencer, 2009; Timmers et al., 2012), several animal studies have shown that they are able to cross the blood–brain barrier. For example, peripheral administration of EGCG (50 mg/kg i.p.) (Lee et al., 2004; Park et al., 2010) and resveratrol (20 mg/kg, i.p.) (Raval et al., 2006; Sakata et al., 2010) exerted neuroprotective actions in rodent models of ischemia (Nagai et al., 2002; Bastianetto and Quirion, 2010) and transgenic models of AD (Rezai-Zadeh et al., 2005; Karuppagounder et al., 2009). Moreover, a significant (one-third) portion of administered [3H]EGCG was found in the mouse brain after a single administration of the radioligand, suggesting that regular consumption of green tea may enable the brain to maintain a fairly high level of EGCG (Suganuma et al., 1998). It is therefore likely that regular consumption of tea (black or green) provides sufficient amounts of phenolic compounds to offer neuroprotection. In support of this hypothesis, various epidemiological studies have reported that tea consumption (2 or more cups /day) reduces (from 28 to 60%) the risk of Parkinson's disease (Checkoway et al., 2002; Tan et al., 2003) and attenuates (up to 43%) the rate of cognitive decline (Ng et al., 2008; Arab et al., 2011), whereas red wine (in moderation) reduces the risk of developing dementia (Orgogozo et al., 1997; Truelsen et al., 2002; Luchsinger et al., 2004; Dai et al., 2006).

The exact mechanisms involved in the activation of PKCγ remain to be established. It has been shown that PKCγ is upstream of several signaling pathways, including ERK and the mammalian target of rapamycin (mTOR) (Menard and Quirion, 2012), which are involved in several neurological disorders, including AD (Klafki et al., 2009; Ma et al., 2010). Interestingly, it has been reported that resveratrol can facilitate cell survival in the ischemic heart by modulating ERK signaling (Das et al., 2006) and decreasing the volume of infarcts in rats (Sinha et al., 2002). It is also known that serum deprivation reduces intercellular communication that can be attenuated by EGCG through ERK activation in endothelial cells (Zhao et al., 2011). In contrast, EGCG restores Aβ-induced cognitive impairments through the inhibition of ERK in mice (Lee et al., 2009). Moreover, opposite effects of polyphenols on the mTOR signaling pathway have been reported. While the inhibition of inflammation by resveratrol in microglial cells requires mTOR activation (Zhong et al., 2012), polyphenols are generally considered inhibitors of this pathway (Menendez et al., 2011). It would thus be worthwhile to explore further the effects of polyphenols on ERK and mTOR signaling following PKC inhibition in neural cells.

Taken together, these results highlight a role for PKCγ in cell survival, maintenance of the cytoskeleton architecture and neuronal plasticity. Interestingly, treatment with polyphenols protects hippocampal neurons against GF 109203X-induced cell death and cytoskeleton degeneration through stimulation of PKCγ phosphorylation. Thus, regular consumption of green tea and red wine, which are enriched in EGCG and resveratrol respectively, may activate neuroprotective signaling pathways, confirming their relevance in preventing age-related neurological disorders.

## Author contributions

Caroline Menard, Stéphane Bastianetto and Rémi Quirion designed research; Caroline Menard and Stéphane Bastianetto performed research; Caroline Menard and Stéphane Bastianetto analyzed data; Caroline Menard, Stéphane Bastianetto and Rémi Quirion wrote the paper.

### Conflict of interest statement

The authors declare that the research was conducted in the absence of any commercial or financial relationships that could be construed as a potential conflict of interest.
